# More than a feeling? What does compassion in healthcare ‘look like’ to patients?

**DOI:** 10.1111/hex.13512

**Published:** 2022-06-03

**Authors:** Sofie I. Baguley, Alina Pavlova, Nathan S. Consedine

**Affiliations:** ^1^ Department of Psychological Medicine, Faculty of Medical and Health Sciences University of Auckland Auckland New Zealand

**Keywords:** compassion, empathy, healthcare, patient preferences, patient–physician relationship, topic modelling, medicine

## Abstract

**Objective:**

Compassion is important to patients and their families, predicts positive patient and practitioner outcomes, and is a professional requirement of physicians around the globe. Yet, despite the value placed on compassion, the empirical study of compassion remains in its infancy and little is known regarding what compassion ‘looks like’ to patients. The current study addresses limitations in prior work by asking patients what physicians *do* that helps them feel cared for.

**Methods:**

Topic modelling analysis was employed to identify empirical commonalities in the text responses of 767 patients describing physician behaviours that led to their feeling cared for.

**Results:**

Descriptively, seven meaningful groupings of physician actions experienced as compassion emerged: listening and paying attention (71% of responses), following‐up and running tests (11%), continuity and holistic care (8%), respecting preferences (4%), genuine understanding (2%), body language and empathy (2%) and counselling and advocacy (1%).

**Conclusion:**

These findings supplement prior work by identifying concrete actions that are experienced as caring by patients. These early data may provide clinicians with useful information to enhance their ability to customize care, strengthen patient–physician relationships and, ultimately, *practice medicine in a way that is experienced as compassionate by patients*.

**Public Contribution:**

This study involves the analysis of data provided by a diverse sample of patients from the general community population of New Zealand.

## 1 INTRODUCTION

*Compassion* has been defined as an emotion,^1^ a motivation^2^ and a virtuous response.^3^ At the least, it involves both feeling and action components^2^, ^4^, ^5^ the *awareness* of suffering and *acting* to alleviate it.^6^ In medicine, compassion is desired by patients, mandated by medical regulatory bodies and increasingly linked to positive outcomes for patients and families, professionals and healthcare systems.^7^ Patients and families rate compassion among the most important healthcare requirements,^8^, ^9^, ^10^, ^11^, ^12^ recalling it years later.^13^, ^14^ Compassionate care predicts faster recovery,^15^ greater autonomy,^16^ lower intensive care utilization^17^ and more responsible healthcare management.^18^ Similarly, compassion‐related constructs have been associated with *objective* benefits, including better disease control^19^ and reduced metabolic complications^20^ among patients with diabetes. Compassion is thus central to both the practice of effective medicine and essential in the preferences of those receiving professional care.

However, despite the value patients place on compassion and the benefits it may have, data circumscribing patients' experiences of compassion are lacking.^21^ Complicating the study of compassion in healthcare is the fact that it has often been confused with other terms, such as empathy, sympathy and concern.^22^ However, while sympathy shares some surface similarities with compassion, sympathy can arise in response to a range of feelings while compassion more specifically arises in response to the *suffering* of another and necessarily includes a motivation to relieve suffering.^3^ Similarly, while empathy is periodically conflated with compassion, empathy does not require action and it may be difficult to sustain over long periods of time.^23^

More to the point, while compassion is experienced as distinct/preferable to empathy or sympathy,^24^ exactly what compassionate care entails or ‘looks like’ to patients is unclear. To date, studies suggest effective communication,^25^ interpersonal connections,^26^, ^27^ understanding,^28^ being present, empathizing,^26^ taking action and providing individualized care^29^ are important to the experience of compassion. Other studies highlight the importance of touch in the experience of compassion,^26^ safety, authenticity and connection.^30^ One study explored how doctors communicate compassion by developing a taxonomy of compassionate physician behaviours in the realm of oncology.^31^ Analyses suggested that the recognition of the patient's suffering, emotional resonance, and movement towards addressing suffering were all important elements in compassion interactions. Of note, these behaviours were experienced as compassion across a conversation rather than in terms of a single event, and silence was associated with emotional resonance. Importantly, patient data suggest that feeling cared for often takes only a moment, while nonpatient views often imply that compassionate care is time‐consuming.^27^, ^32^ It is also possible that compassion may be experienced differently between the healthcare provider expressing compassion, the patient receiving it, or others observing the interaction. Nonetheless, evidence to date suggests that patients experience care when practitioners are emotionally present, communicate effectively, enter into their experience and display understanding and kindness. While these factors are clearly important to patients, what physicians might actually *do* to create the experience of care remains unknown.

More broadly, there are at least three significant limitations to prior studies of the patient experience of care. First, most patient studies have been conducted in nursing contexts^18^, ^26^, ^33^ or palliative care samples.^21^, ^24^, ^34^ While such contributions are important, findings may be less applicable to *general* patient samples. For example, caring behaviours are often thought to be ‘part and parcel’ of nursing,^35^ creating the possibility that behaviours from different professionals in different contexts may be experienced in different ways. Equally, it is unclear whether perspectives from palliative care will translate to health contexts where patients have distinct clinical and personal priorities.^36^

Second, prior studies have concentrated on the patients' *experience* of care rather than on what physicians should do to engender this experience. While imperative to understanding compassion, studying a patient's experience does not provide direct clinical or educational guidance because it implies that physicians should behave in ways that generate an outcome (the feeling of being cared for) rather than identifying the behaviours themselves. Finally, to this point, studies investigating patient perspectives on compassion have been derived from *qualitative* data and in modestly sized samples and used a single tool of either patient experience or patient evaluation. In the current report, a dual experience/evaluation approach was used that validates the clinical utility of such data.^37^ Additionally, an alternative approach to text analysis that combines machine learning techniques with text‐based interpretation^38^ by looking at ‘vocabularies’ or probabilistically co‐occurring words^39^ is undertaken. Although traditional qualitative analyses by coding may richly characterize what constitutes compassionate care in the patient's eyes, the coding of responses may introduce researcher bias.^40^ Specifically, the risk with such designs is that in creating and refining coding systems, researchers may (involuntarily) impose their own beliefs, knowledge and interests, which may (or may not) reflect patient meaning regarding compassion. While text analysis also has limitations^41^ such as not being able to interpret latent context (e.g., humour, irony or polysemes), this approach can reveal unbiased themes as well as themes that researchers might not notice or code for to deepen our understanding of compassionate care.

In contributing to this nascent area of study, the current report presents data from a large sample of community‐based patients, identifying the physicians' actions that are seen as characterizing caring behaviour for patients.^42^ In shifting the focus from patients' experiences of care to identifying physicians' actions that communicate compassion and using an analytic framework that avoids some forms of researcher bias, the current study addresses several limitations in the prior research of compassionate care and outcomes related to patient–physicians relationships interventions.^43^ Findings can thus supplement existing work in helping to identify the physician's actions that matter to patients and thus offer clinicians an initial glimpse at a future compassion tool kit with the potential to enhance their ability to customize care, strengthen the patient–physician relationship and, ultimately,  *practice in ways that are experienced as caring*.

## 2 METHODS

### 2.1 Study design

Data for this report were taken from a broader study of compassion in healthcare in 1065 community patients and 219 of their physicians in New Zealand. Ethics approval was obtained from the University of Auckland Human Participants Ethics Committee on 17 June 2020 (Approval Number: 024749). To be eligible to participate, patients needed to be 18+ years, English speaking and have a physician they had seen for 3+ clinical visits to ensure an established (versus new) patient–physician relationship.

### 2.2 Procedure

The study was advertised via social media postings, email lists and word‐of‐mouth. Given the potential sensitivity of patient data, community participants were anonymous and data submission was taken as consent. A link directed prospective participants to an information sheet and consent form. Following consent, demographic, healthcare utilization and health information were gathered before specific questions about the relationship and experience of compassion with their physician were delivered.

### 2.3 Measurement

*Compassionate care*. In line with the primary research question, patients were asked to describe their experience of compassionate care with their physician. Patients were provided with a brief definition of compassion (*Compassion in medicine is the ability to recognize and understand a patient's suffering, coupled with the desire to relieve it*) before being asked a single yes/no item to the question: ‘do you feel your physician cares for you and wants to help?’ if patients selected yes, they were asked to describe specifically what their physician does that made them feel cared for.

### 2.4 Analyses

First, the text was cleaned in the Python (3.10) programming language^44^ by removing stop words (e.g., and, or, that). Bi‐grams and tri‐grams were created to account for phrases. Second, data were analysed using Latent Dirichlet Allocation (LDA) topic modelling (TM).^45^ This inductive quantitative technique searches for latent structures by clustering words with a higher probability of co‐occurring in texts than expected to happen by chance.^46^ Since LDA modelling arranges these latent structures (or vocabularies) proportionally,^47^ we can identify which physician caring actions are referred to more frequently and are hence of greater importance to patients. As an alternative to more traditional qualitative thematic analysis, TM is more robust in application to larger data sets and helps avoid the (involuntary) imposition of researcher bias. The TM analysis was conducted via one of the most widely used tools—Machine Learning for Language Toolkit (MALLET) for Mac,^48^ which is considered best in class due to precision in sampling methods.^49^

**Figure 1:**
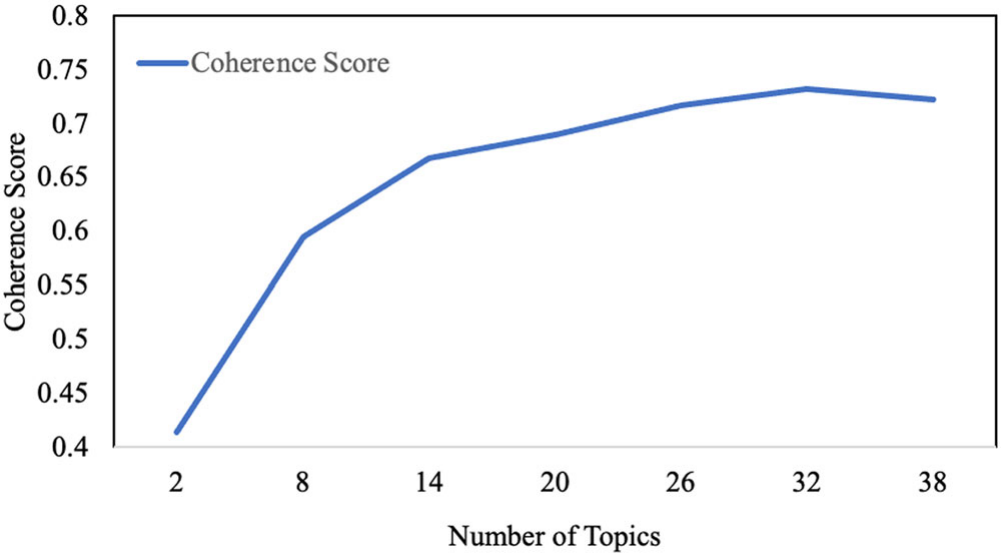
Coherence growth stabilization curve

The number of topics was determined based on the coherence score. Coherence scores can be defined as the ease with which topics can be interpreted by taking a median of pairwise word‐similarity scores within a given topic for a group of topics.^50^ Hence, to develop the topic solution best fitted to the data, a range of LDA models with different topic numbers (*k*) was built in a single algorithm. In a manner similar to scree plot interpretation, the number of topics is chosen based on the first flexion point, indicating coherence growth stabilization (see Figure 1). The standard default number of keywords in MALLET is set to 20.

After topic numbers are determined, the number of texts per topic is manually assessed by the research team to develop appropriate topic labels. Each response is assigned a single dominant topic based on the highest topic proportion (prominence) per document. Final topics proportions were calculated based on dominant topics and aligned with the model's output *α* values that denote the total topic distributions over documents.^51^, ^52^

## 3 RESULTS

### 3.1 Participants

Seventy‐two percent (767/1065) of participants responded to the question regarding physician compassionate care. Patients ranged in age from 18 to 82 years (*M* = 41.87, SD = 14.85), 91% identified as female and 1% as gender‐diverse. Most identified as NZ European (73%), followed by NZ Māori (12%), Asian (5%), Pasifika (3%) or other (18%). More than two‐thirds of the participants (71%) reported having been diagnosed with one or more serious or chronic health conditions, including heart disease (10%), gastrointestinal (bowel) problems (15%), (auto)immune conditions and endocrine disorders (15%), cancer (7%), mental health concerns (35%) and other serious or chronic health concerns that the participants self‐identified (e.g., asthma, arthritis, chronic pain, diabetes, eczema etc.) (39%). Nearly nineout of ten participants made ratings regarding a primary care physician, with others rating either a specialist (2%) or other types of nonspecialized practitioners (e.g., nurse, psychologist, counsellor etc.) (2%) (see Table 1).

**Table 1:** Analyses

Variables	*Did not answer the care question* (*N* = 108)	*Patients reported feeling their physician does not care* (*N* = 147)	*Patients reported care but did not explain how* (*N* = 43)	*Patients reported feeling cared for and explained how* (*N* = 767)
Mean age (SD)^1^	38.19 (15.5)^a^	40.45 (14.08)	41.02 (14.97)	41.87 (14.86)^b^
*N* (%) female^2^	68 (88%)	137 (93%)	36 (83%)	700 (92%)
Ethnicity^2^				
NZ European	60 (78%)	103 (70%)	31 (74%)	581 (76%)
NZ Māori	7 (9%)	17 (12%)	5 (12%)	93 (12%)
Asian	5 (7%)	8 (5%)	4 (10%)	33 (4%)
Pasifika Peoples	3 (4%)	5 (3%)	1 (2%)	27 (4%)
Other	10 (13%)	34 (23%)	7 (17%)	133 (17%)
Diagnosed with serious or chronic illness^2^	10 (9%)^a^	94 (64%)	23 (53%)^b^	549 (71%)^a,b^
Heart	1 (1%)^a^	11 (7%)	5 (12%)	80 (10%)^a^
Bowel	4 (4%)^a^	17 (12%)	4 (9%)	106 (15%)^a^
Immune	4 (4%)^a^	26 (17%)	5 (12%)	112 (15%)^a^
Mental health	4 (4%)^a^	42 (29%)	14 (33%)	270 (35%)^a^
Cancer	0 (0%)^a^	1 (1%)^b^	1 (2%)	26 (7%)^a,b^
Other serious chronic illness	5 (5%)^a^	56 (38%)	11 (26%)	296 (39%)^a^
Type of physician^2^				
Primary care	–	100 (68%)^a^	32 (75%)	674 (88%)^a^
Specialist	–	4 (3%)	0 (0%)	17 (2%)
Other	–	5 (3%)^a^	1 (2%)	11 (2%)^a^

*Note*: Means or percentages with the same superscript differ from one another at *p* < .05.

^1^ Tested via independent samples *t*‐tests.

^2^ Tested by *χ* ^2^ test.

Testing the group of patients who reported feeling cared for and provided a text description from the other groups showed some differences. They did not differ from other groups in terms of ethnicity or gender (*p* > .05). However, people who did not answer the care question were younger than the group of primary interest, *t*(842) = 2.075, *p* < .05, although the effect size was small, *d* = 0.23. They were also less likely to be diagnosed with any serious chronic illness (*χ* ^2^[1] = 159.35, *p* < .01, odds ratio [OR]: 24.68) and consistently did not report their doctor's specialization. In comparison to the people who did not find their doctor caring, patients who reported feeling cared for and described how their doctor cared were also more likely to be diagnosed with cancer (*χ* ^2^[1] = 8.61, *p* < .05, OR: 10.84); they were more likely to make ratings regarding a primary care physician (*χ* ^2^[1] = 3.95, *p* < .05, OR: 2.17) and less likely to rate another type of nonspecialized practitioner (nurse, psychologist, counsellor) (*χ* ^2^[1] = 4.45, *p* < .05, OR: 0.33). People who reported feeling cared for but did not provide a written reflection were less likely to be diagnosed with any serious chronic illness, although with a very small OR (*χ* ^2^[1] = 6.42, *p* < .05, OR: 0.46).

The primary TM analysis revealed eight topics within the texts describing physician behaviours leading to patients feeling cared for. Seven topics were coherent and could be labelled, the eighth could not. In order of their commonality, topics were: listening and paying attention to the patient (71% of texts), following‐up and running tests (11%), continuity and holistic care (8%), respecting preferences (4%), genuine understanding (2%), body language and empathy (2%) and counselling and advocacy (2%) (see Table 2).

**Table 2:** LDA‐derived topics describing what physicians do to express care according to patients (*N* = 767, *T* = 8)

(A)											
Topic number	Topic name	Keywords	Alpha	Dominant topic % of documents	Age (mean/SD)	% Female	New Zealand European/Pākeha	Māori	Pacific Peoples	Asian	Other
1	**Listening and paying attention to the patient**	Feel time questions asks listens things health life concerns listen makes takes family care issues treatment caring make doctor good.	1.12	70.7%	42 (14.87)	91%	74.90%	13.10%	3.50%	4.60%	18.10%
2	**Deliverables**	Tests check follows_up results follow_up appointments calls test phone back extra call blood home appointment sick questions referrals concerned quick.	0.24	10.7%	46 (14.99)^a^	93%	80.50%	7.30%	2.40%	1.20%	19.50%
3	**Continuity and holistic care**	Medical doctor care asked felt family listened referred cares great mother cared visit practice ago wonderful times years email child.	0.20	8.2%	41 (14.93)	94%	82.50%	6.30%	0%	4.80%	15.90%
4	**Respecting preferences**	Refer patient prescription medication office specialists situation giving decisions computer deal unsure messages alternative preferences fantastic speaks back ideas learn.	0.10	3.8%	38 (15.35)	93%	69.00%	20.70%	6.90%	3.40%	13.80%
5	**Genuine understanding**	Pain genuinely seeking difficult aware appt believes amazing straight urgent investigation showing nice practitioner positive friendly helped level dismissed rushed.	0.07	2.0%	47 (14.49)	73%^b^	93.30%	0.00%	13.30%^b^	0.00%	13.30%
6	**Body language and empathy**	Contact language body speak eye make sti lots competent bit stupid human community midwife input collaborative simple proactive sad for example	0.07	1.7%	42 (14.84)	92%	61.50%	23.10%	15.40%^b^	7.70%	15.40%
7	**Counselling and advocacy**	Chronic relevant feeling hospital found script depression words advocate neuritis vestibular PCOS sorts woman constantly counselling sensitive stress healthy told.	0.05	1.5%	34 (9.51)^a^	92%	83.30%	8.30%	0%	16.70%^b^	8.30%
8	*Not labelled*	Diagnosis cancer specialist years health answering journey mum ear current put validates recovery sound listeners bowel polyps entire bleeding me/my.	0.04	1.4%	40 (13.31)	82%	72.70%	18.20%	0%	0%	18.20%

(B)								
**Topic number**	**Topic name**	**Keywords**	***α***	**Dominant topic % of documents**	**Serious/chronic illness diagnosis (%)**	**GP**	**Specialist**	**Other HCP**
1	**Listening and paying attention to the patient**	Feel time questions asks listens things health life concerns listen makes takes family care issues treatment caring make doctor good.	1.12	70.7%	69.1%	87.0%	2.4%	2%
2	**Deliverables**	Tests check follows_up results follow_up appointments calls test phone back extra call blood home appointment sick questions referrals concerned quick.	.24	10.7%	80.5%	89.0%	3.7%	0%
3	**Continuity and holistic care**	Medical doctor care asked felt family listened referred cares great mother cared visit practice ago wonderful times years email child.	.20	8.2%	68.2%	95.0%	0%	0%
4	**Respecting preferences**	Refer patient prescription medication office specialists situation giving decisions computer deal unsure messages alternative preferences fantastic speaks back ideas learn.	.10	3.8%	86.2%	90.0%	3.4%	0%
5	**Genuine understanding**	Pain genuinely seeking difficult aware appt believes amazing straight urgent investigation showing nice practitioner positive friendly helped level dismissed rushed.	.07	2.0%	86.7%	93.0%	0%	0%
6	**Body language and empathy**	Contact language body speak eye make sti lots competent bit stupid human community midwife input collaborative simple proactive sad for example.	.07	1.7%	84.6%	100%	0%	0%
7	**Counselling and advocacy**	chronic relevant feeling hospital found script depression words advocate neuritis vestibular PCOS sorts woman constantly counselling sensitive stress healthy told.	.05	1.5%	75%	67%	0%	0%
8	*Not labelled*	diagnosis cancer specialist years health answering journey mum ear current put validates recovery sound listeners bowel polyps entire bleeding me/my.	.04	1.4%	63.6%	100%	0%	0%

Abbreviations: HCP, healthcare professional; LDA, Latent Dirichlet Allocation; PCOS, polycystic ovary syndrome.

^a^ Tested via independent samples *t*‐tests; significant at *p* < .05.

^b^ Tested by *χ* ^2^ test; significant at *p* < .05.

### 3.2 Topics representing compassionate care

#### 3.2.1 Topic 1: Listening and paying attention to the patient

A common thread throughout all of the reflection, and being dominant in 71% of the patient accounts, physicians' ‘listening and paying attention to the patient’ dominated the text. Patients reported that compassionate physicians are ‘generally […] good listeners’ and display ‘listening with the intention to help support and help […] articulate exactly what it is that [the patient is] trying to explain’. As summarized by one patient ‘(I) feel looked after when (physicians) listen and seem to care, they remember (patient's) name, they give […] time while treated, […] answer questions’. Other patients stated that it is important for physicians to ask questions about their illness, talk about general life, inquire about family, take an interest in a person and make him/her comfortable to discuss anything. Overall, physicians listening, asking questions and paying attention appear to be a central part of the experience of care.

#### 3.2.2 Topic 2: Deliverables

‘Deliverables’ were the second most frequently reported indication of physician care. Responses included experiencing compassion when physicians are ‘following‐up and running tests’, which featured at least once in 24% of the reflections and was a dominant topic in 10.7% of the texts. Most reflections categorized under this topic mentioned physicians following up with patients to explain test results, usually by phone or email, and checking how the patient was feeling after an appointment. Additionally, ‘following up’ often succeeded ‘running necessary tests’. Hence, patients experience compassion when their physicians ‘follow up with a call or text a day or 2 after the appointment’, ‘ring […] to discuss results’, ‘refer when necessary’ and ‘carry out tests required (e.g., blood tests)’, including screening tests. Patients whose narratives reflected the importance of deliverables were significantly older than the average. The difference of 3.99 years (95% confidence interval [CI]: [0.7, 7.3]), was significant *t*(81) = 2.412, *p* < .05; however, the effect size was small, *d* = 0.27.

#### 3.2.3 Topic 3: Continuity and holistic care

The theme ‘continuity and holistic care’ featured in 20% of the reflections and was a dominant topic in 8% of the documents. While comments were concentrated on continuity of care (perhaps indicating the importance of long‐term relationships to compassion), statements contributing to this topic also referenced preventative/holistic care, notably in the context of dealing with chronic and complex illnesses and going ‘above and beyond’. For instance, one patient reported ‘He has been my doctor for many years and is my children's doctor, my daughter had leukaemia… he would personally call us after hours to check on us etc., he also growls us if not looking after ourselves. He actually cares’.

Of note, most of the reflections in this category mentioned family care (*have been our families doctor for over 22 years*), long‐term management of chronic or complex illness and emphasizing preventative/holistic care (*my doctor considers alternative therapies alongside conventional therapies, he is all about prevention rather than treatment!, suggesting things that could help me in my lifestyle*). Other patients referred to their physicians going the extra mile *‘*calls outside of hours, late and night and during weekends. Goes over appointment times to make sure everything is sorted/nothing is missed’*. *‘He doesn't need prompting on my medical history, he remembers exactly where we left off. He always replies to my emails. He knows my family and family situations and has a great sense of humour’.

#### 3.2.4 Topic 4: Respecting preferences

‘Respecting preferences’ represented 10% of patient accounts and was dominant in 4% of the texts. Most of the reflections on this topic were centred around patients feeling that their preferences were respected and being actively involved in decision‐making *(he is respectful of my perceptions and preferences and need to be an active participant in my own healthcare, gauges which treatment option sits right with me, listen to my alternative ideas for treating long term condition and accepts my reluctance to* ‘over medicate’). Acknowledging one's preferences and showing respect incorporated commentary regarding physician cultural awareness *(culturally aware and educated and speaks to me about research and my ethnic group and statistics, we have a conversation, where he says his korero, I have a korero, it is informal, relaxed, I learn more about my illness from each visit*) and general respect *(doesn't look at his computer*).

#### 3.2.5 Topic 5: Genuine understanding

The fifth topic represented 7.0% of the documents and was dominant across 2% of the documents. The main themes revolved around patients feeling validated and believed by physicians (*they genuinely believe that i'm sick*) seeking understanding and investigating symptoms (*seeking to fully understand the situation, seeking solutions, attempts to identify the cause[s]*). This topic also incorporated the importance of being genuine *(she genuinely ‘has my back’, makes no nonsense, honest, genuine, informed and kind*), positive and genial nature of the physician. Conversely, physicians were not considered caring when they were experienced as rushed, rude or dismissive (*seem rushed and sometimes are blunt and a bit rude, have dismissed an experience of persistent or repeated pain*). Based on these reflections, the fifth topic was labelled ‘genuine understanding’. The patients who valued genuine understanding were more likely to identify as males (*χ* ^2^[1] = 7.09, *p* < .05, OR: 3.94) and be of Pasifika descent (*χ* ^2^[1] = 4.50, *p* < .05, OR: 4.11).

#### 3.2.6 Topic 6: Body language and empathy

The topic ‘body language and empathy’ represented 7.0% of the documents and was dominant in 2% of the statements. Patients reported feeling cared for when their physician uses body language or empathetic gestures *(e.g., moves his chair to face me, may make physical contact)*. Smiling, nodding, facial expressions, not speaking over the patient, using simple language, holding a hand—all were examples of small compassionate acts that contribute to a patient's experience of care. Importantly, body language was frequently used in conjunction with descriptors, such as empathetic, *compassionate*, interested, welcoming and engaged. The patients who valued body language and empathy were more likely to be of Pacific descent (*χ* ^2^[1] = 4.68, *p* < .05, OR: 4.93).

#### 3.2.7 Topic 7: Counselling and advocacy

The seventh topic labelled ‘counselling and advocacy’ represented 5% of the documents and was dominant in 2% of the documents. In this topic, patients referred to chronic illness (*polycystic ovary syndrome, migraines*), mental illness (*depression, post‐traumatic stress disorder*), trauma and diagnoses with somatic complaints. Doctors were said to be non‐judgemental towards such concerns, showing sensitivity and using interventions, such as counselling, mindfulness and advocating for the patient. As one patient recalled, ‘she even notes these things if they're relevant to any referrals I need, saving me from having to constantly advocate for myself’. The patients who found counselling and advocacy important were significantly younger than the average. The difference of 8.37 years (95% CI: [−14.41, −2.33]), was significant *t*(12) = −3.049, *p* < .05 and was of medium‐to‐large effect size, *d* = 0.67. These patients were also more likely to be of Asian descent (*χ* ^2^[1] = 4.50, *p* < .05, OR: 4.29).

Finally, the last topic that characterized 5% of the documents, but was only dominant in 1% of the documents, was difficult to discern. Most of the reflections overlapped with other topics. The two most illuminating reflections in this account spoke about diagnosis, particularly misdiagnosis, which might hint at what the topic could have entailed if greater data were available. The repetition of the keyword validation is also important to note.

## 4 DISCUSSION

Although compassion is central to both patient and physician perspectives and values in healthcare, the empirical base regarding compassion is modest. More specifically, while it has been clear that patients value the experience of compassion, prior work has typically been conducted in particular contexts (e.g., palliative care) and/or studied the experience of care in ways that have not offered guidance to physicians regarding how to behave in ways that communicate care. In contributing to this nascent area of work, this report identified seven elements of physician behaviour and interaction contributing to patients' experience of compassion: listening and paying attention to the patient (71%), following‐up and running tests (11%), continuity and holistic care (8%), respecting preferences (4%), genuine understanding (2%), body language and empathy (2%) and counselling and advocacy (2%). Below, the implications these findings have for research and clinical practice are considered in greater detail, findings are reintegrated with prior research and consideration is given to the importance of action to compassion for patients. Study limitations and future directions are discussed.

The study revealed significant differences according to age and ethnicity. While direct causations cannot be inferred, some reflections on what may contribute to the present findings are offered below. Regarding age, younger participants were less likely to respond to the question asking about experiences of compassion with their physician, report on the type of specialization of their physician and have a diagnosed health condition. This may index less frequent interactions within healthcare among younger, healthier samples, who might not reflect as much on their experiences with their physician. Additionally, younger patients were more likely to report the importance of counselling and advocacy in the experience of compassion and were also more likely to be of Asian descent. This pattern might suggest that younger patients value explanations, sensitivity and advocating with relevant referrals. In Asian cultures, where debilitating illness and mental health challenges are associated with high stigma,^53^ particular elements of care, sensitivity and being nonjudgemental may enhance the patient–physician relationship and the experience of interactions as compassionate. Interestingly, patients who reported ‘deliverables’, including follow‐up, running tests and phone calls, were significantly older. This may reflect more complex health needs requiring follow‐up or the importance older adults may place on engagement and follow‐through. Finally, patients who valued body language and empathy and genuine understanding were more likely to be of Pasifika descent. Ethnographic data suggest that Pasifika cultures place high importance on trust and rapport building and value the va (space between places or people) to connect in mutual respect.^54^ Our data may suggest such values may also be reflected in their experiences of compassion. While underrepresented in the present study, males reported the importance of genuine understanding with comments about feeling ‘understood or believed’. Overall, the data indicate that sociodemographic characteristics, including age and ethnicity, may contribute to differences in the factors patients experience as comprising compassionate care. Further work is necessary to explore these factors in more depth.

In extending compassion research more broadly, this study provides three core contributions. First, patient perspectives of compassion in a large, diverse and general population were examined using an analytic approach that supplements prior methodologies.^45^ As noted, most studies of patient perspectives have been conducted in modestly sized samples and restricted to nursing and palliative care,^21^ contexts that may have particular needs^55^, ^56^ regarding how care is best expressed. In studying compassion in the general population (*N* = 767), these data represent the views of patients in an established physician relationship, with a range of health conditions and concerns, and from diverse ethnic and developmental backgrounds; these data thus enhance generalizability to broader samples.

Prior work has employed traditional thematic analysis to explore patient perspectives of compassion using theoretical frameworks,^17^, ^57^ potentially constraining patient perspectives and introducing researcher bias. In contrast, the machine learning techniques (topical modelling) used in the current report allow for the identification of themes from qualitative responses using text categorization and opinion mining without imposing a priori themes onto the data.^58^ TM revealed seven coherent topics within patient responses, representing specific physician actions seen as communicating compassion by patients.

In some ways, the findings from these analyses are broadly consistent with earlier work, offering a methodologically robust supplement and confirmation to what has been seen previously. Descriptively, the core topic in 71% of responses referred to listening and paying attention to the patient, suggesting these characteristics are central (or necessary) to the experience of compassion in healthcare.^59^, ^60^, ^61^ Prior studies suggest that patients rate active listening and paying attention among the most important qualities in a competent physician.^62^ Authentic listening allows patients to feel seen, strengthens relationships and facilitates a healing process.^63^ Compassion may lead to better outcomes because listening and attending promote trust and disclosure and thus more accurate diagnoses.^64^

However, while listening and paying attention to the patient might be essential to the experience of care, the tendency of prior studies to focus on this characteristic^12^, ^26^, ^34^ has obscured the examination of what *actions* are interpreted as care. Moreover, the data from this study suggest that ‘listening and paying attention to the patient’ is a large topic that is rather vague and broad, and is generally in line with the definition of compassion in a sense of being seen and heard.^65^ While we acknowledge the possibility that listening and paying attention to the patient may or may not be a unitary construct in patient experience and that further work is needed, as it has been derived from other topics, the experience of compassion in patients also requires *more than active listening* that invokes a feeling.^26^ Rather, patients experience concrete actions involving following up and proactivity in their care *as compassion*. Indeed, the second most frequently reported topic reflected health ‘deliverables’, concrete behaviours such as following up and running tests *as indexing physician compassion*. These data suggest that having their physician call or message to advise them of test results, updates or bookings was experienced as a crucial part of caring. This observation also aligns with the fact that earlier studies have suggested that following through is important and taken as a sign of a physician's excellence.^66^

In addition to doctors' following up, patient commentary suggested that continuity of care, openness to holistic practices, respecting preferences, and expressing genuine concern mattered. Each of these actions has been alluded to in prior studies,^66^, ^67^, ^68^, ^69^, ^70^, ^71^, ^72^ although it has been unclear whether actions of this kind are separate elements of care or part of a general approach that patients are responding to. Given these areas showed up in different *topics*, the analyses presented here suggest that the behaviours that communicate care to patients are diverse, encompass multiple elements and, more speculatively, may imply that different patients have a distinct ‘language of care’.

In other regards, however, our findings were somewhat at odds with prior evidence. Previous studies have suggested that a physician's body language and empathetic demeanour are important for the experience of care via associations with greater patient satisfaction,^73^, ^74^ trust and partnership,^75^ adherence,^76^, ^77^ reduced anxiety and depression in palliative care samples^78^ and better overall clinical outcomes.^19^, ^79^, ^80^ However, the present study found compassionate acts, including body language, empathy and gestures (e.g., kindness, showing interest, physical touch and using simple language) only accounted for 1.7% of texts describing compassionate behaviour. Because patients may be more likely to use language or terms that do not highlight empathy per se, it may be that the variance typically associated with empathy is being captured by patient texts highlighting active listening and attentiveness. Alternately, it may be that empathy is less important to the experience of care than previously thought.

## 5 LIMITATIONS

While the present study extends our understanding of compassion from the patient's perspective, it is not without limitations. First, the sample was predominantly female, a common limitation of digital recruitment with medical internet studies, indicating this may be due to fewer men responding to studies and recruitment in general,^81^ and a higher percentage of females using Facebook^82^—the predominate method of recruitment in the present study. Although we are not aware of studies in compassion specifically, the possibility that there are important gender differences in the normative languages of care seems likely and would be a fertile area for future study. Additionally, advertising via social media postings and email listings may have excluded individuals without access to the internet, contributing to selection bias. Second, while analyses suggested few differences in age, ethnicity, gender and diagnosis between our sample and the parent study, power is likely an issue and further testing of how the behaviours that communicate care might vary across groups is clearly warranted. Third, while TM analysis is a highly robust sampling method for large data sets and employs a relatively atheoretical framework, it is unable to interpret latent content, potentially reducing the accuracy of some interpretations.^41^ Additionally, it is useful to note that patients were first provided with a definition of compassion before being asked ‘do you feel your physician cares for you and wants to help? Ultimately then, we are inferring the perception of the doctor as compassionate based on the extent to which patients felt cared for. Whether there is a distinction between these two concepts is an interesting possibility that has not been studied empirically. While this may be seen as a limitation of the present study, this method had the advantage of minimizing conflation with empathy, using everyday language suited to a general sample, and reducing the risk of desirability bias. Finally, it should be remembered that it is likely a combination of ‘strategies’ that communicate compassion to patients rather than a single (even if most prominent) ‘strategy’ being needed. Nonetheless, having identified a more precise portrait of actions of care, future research may test each of the seven core themes revealed here in a larger sample, more deeply consider the meaning of topics via focus groups and develop instrumentation to facilitate evaluations of how each topic might contribute to the experience of care.

## 6 CONCLUSION

Compassion is desired by patients, professionally mandated and central to effective clinical care, with potential benefits throughout the healthcare system. Yet, despite its importance, the physician behaviours that communicate compassion to patients have remained unclear, with prior work concentrated on the *experience* of care. The present study employed a mixture of quantitative and qualitative techniques to contribute to knowledge in this area, revealing key themes constituting the experience of care from the patient's perspective. Taken as a whole, this study confirms and extends prior work in a large, diverse sample of patients. Perhaps most importantly, our analyses suggest that *compassion is more than just a feeling* for patients and that there is a range of *concrete techniques* that physicians may engage in, which are normatively experienced as compassionate by patients. Further work focusing on real, concrete skills or behaviours will inform the development of targeted interventions and training to enhance the experience of compassionate care.

## CONFLICTS OF INTEREST

The authors declare that the research was conducted in the absence of any commercial or financial relationships that could be construed as a potential conflicts of interest.

## ETHICS STATEMENT

The studies involving human participants were reviewed and approved by the Human Participants Ethics Committee, University of Auckland. The patients/participants provided their written informed consent to participate in this study.

## Data Availability

The datasets presented in this article are not readily available because participants consented to participate with the understanding that access to data would be restricted to the named researchers. Requests to access the datasets should be directed to Nathan S. Consedine, n.consedine@auckland.ac.nz.
